# Auto-classification of biomass through characterization of their pyrolysis behaviors using thermogravimetric analysis with support vector machine algorithm: case study for tobacco

**DOI:** 10.1186/s13068-021-01942-w

**Published:** 2021-04-27

**Authors:** Chao Yin, Xiaohua Deng, Zhiqiang Yu, Zechun Liu, Hongxiang Zhong, Ruting Chen, Guohua Cai, Quanxing Zheng, Xiucai Liu, Jiawei Zhong, Pengfei Ma, Wei He, Kai Lin, Qiaoling Li, Anan Wu

**Affiliations:** 1 Fujian Provincial Key Laboratory for Theoretical and Computational Chemistry, College of Chemistry and Chemical Engineering, Xiamen University, Xiamen, 361005 Fujian China; 2 Technology Center, China Tobacco Fujian Industrial Co., Ltd, Xiamen, 361021 Fujian China

**Keywords:** Thermogravimetric analysis, Machine learning, SVM algorithm, Tobacco

## Abstract

**Background:**

During the biomass-to-bio-oil conversion process, many studies focus on studying the association between biomass and bio-products using near-infrared spectra (NIR) and chemical analysis methods. However, the characterization of biomass pyrolysis behaviors using thermogravimetric analysis (TGA) with support vector machine (SVM) algorithm has not been reported. In this study, tobacco was chosen as the object for biomass, because the cigarette smoke (including water, tar, and gases) released by tobacco pyrolysis reactions decides the sensory quality, which is similar to biomass as a renewable resource through the pyrolysis process.

**Results:**

SVM algorithm has been employed to automatically classify the planting area and growing position of tobacco leaves using thermogravimetric analysis data as the information source for the first time. Eighty-eight single-grade tobacco samples belonging to four grades and eight categories were split into the training, validation, and blind testing sets. Our model showed excellent performances in both the training and validation set as well as in the blind test, with accuracy over 91.67%. Throughout the whole dataset of 88 samples, our model not only provides precise results on the planting area of tobacco leave, but also accurately distinguishes the major grades among the upper, lower, and middle positions. The error only occurs in the classification of subgrades of the middle position.

**Conclusions:**

From the case study of tobacco, our results validated the feasibility of using TGA with SVM algorithm as an objective and fast method for auto-classification of tobacco planting area and growing position. In view of the high similarity between tobacco and other biomasses in the compositions and pyrolysis behaviors, this new protocol, which couples the TGA data with SVM algorithm, can potentially be extrapolated to the auto-classification of other biomass types.

**Supplementary Information:**

The online version contains supplementary material available at 10.1186/s13068-021-01942-w.

## Background

Pyrolysis of biomass is a potential method to produce various gases, liquids (bio-oil), or solid materials (bio-char) that can then be used for fuel production. The product compositions depend mainly on the variability of different proportions of protein, triglycerides, hemicellulose, cellulose, lignin, etc., in the original biomass [1, 2]. Therefore, many studies focus on studying the association between biomass and bio-products [3–9]. In this study, tobacco was chosen as the object for biomass. As a commercial product, the cigarette smoke (including water, tar and gases) released by tobacco pyrolysis reactions can satisfy the consumer's demand, not the tobacco itself, which is similar to biomass as a renewable resource through the pyrolysis process.

Tobacco leaves cultivated in different areas have different styles, and their grades are based on the positions they grow on the stalk. The classification of tobacco style and grade is important in the processes of tobacco blend design and cigarette product maintenance [10]. Current evaluation of tobacco style and grade mainly relies on artificial sensory analysis, which is subjective and relatively unstable [11]. Therefore, it is necessary and urgent in the tobacco industry to develop a new rapid and convenient method to evaluate the tobacco style and grade automatically.

Artificial intelligence has opened a new page in the field of data analysis. Many efforts have been devoted to developing automatic evaluation methods using the advanced machine learning (ML) algorithms with the data from the tobacco leaves and smoke. Early works mainly focused on the classification of tobacco cultivation area and growing position using near-infrared spectra (NIR) due to its high efficiency and non-destructive characteristic. Hana et al*.* [12] employed artificial neural networks (ANNs) to classify whether the burley tobacco grows in USA or outside USA, and obtained high prediction accuracy. For the classification of tobacco style and grade, Ni et al*.* [13] developed an improved and simplified K-nearest neighbor algorithm (IS-KNN) to discriminate more than 1000 Chinese flue-cured tobacco leaf samples with moderate accuracy. Their results suggest that it is better to establish a classification model of tobacco grade from the same cultivation fields to get better classification results. By applying a combined random-forest (CRF) based on gas chromatography (GC) fingerprinting, Lin et al*.* [14] managed to classify three different grades of “Furong” series cigarettes with accuracy up to 93.74%. Based on image processing on tobacco color, texture, and shape, Zhang and Zhang [15] implemented a two-level fuzzy comprehensive evaluation (FCE) and classified the tobacco leaves into three grades, but accuracy is achieved just 72% for the non-trained tobacco leaves. Recently, Gu et al*.* [16] successfully built a relationship between chemical compounds and the aromatic quality of flue-cured tobacco leaves, using support vector machine (SVM) algorithm with 22 chemical compounds selected by Relief-F-particle swarm optimization (R-PSO), and obtained high accuracy of 90.95%. Very recently, Wang et al*.* [17] employed genetic algorithm (GA) to optimize the performance of SVM for data analysis of NIR spectroscopy sensors. They demonstrated that the GA could indeed improve the performance of SVM for tobacco classification based on NIR spectra, although the accuracy is just 83%. All previous works have focused on the relationship of tobacco style and grade with either the reactant (tobacco) component or the product (smoke). In this study, we choose to pay attention to the tobacco pyrolysis reaction process, which can be visually expressed by the thermogravimetric analysis (TGA). To the best of our knowledge, the auto-classification of tobacco planting area and growing position based on thermogravimetric analysis have not yet been reported.

TGA has been proven to be a useful tool to study the pyrolysis behavior and kinetics of the pyrolysis process, since it provides precise measurement depending on temperature and other experimental conditions that are well known and well controlled [18–20]. Investigations on biomass have shown that the differences in pyrolytic characteristics are mainly caused by the differences in the constituent and physical structure [21–27]. Studies on the pyrolysis of tobacco have also demonstrated that the differential thermal gravity (DTG) curve of tobacco pyrolysis can be divided into different Gaussian peaks representing the thermal decomposition of individual components [28, 29]. For instance, the mass loss below 373 K represents the evaporation of water [30]; the peaks between 373 and 473 K correspond to the thermal decomposition of sugars, nicotine, pectin, and some other volatile species [31, 32]; and in the temperature of 474-873 K, the mass loss would be attributed to the pyrolysis of hemicellulose, cellulose, and lignin, respectively [33–35]. Moreover, Baker and Bishop [36] have demonstrated that the thermogravimetric analysis spectra of tobacco pyrolysis are highly reproducible under well-defined conditions. The thermogravimetric analysis data not only represent the tobacco pyrolysis characteristics, but also supply the information of the tobacco composition. Hence, it can be taken as an important index to evaluate tobacco planting area and growing position.

Recently, we [37] demonstrated that thermogravimetric analysis data in conjunction with the normalized root-mean-square error (NRMSE) can be used to quantitatively evaluate the pyrolysis difference among tobacco of different stalk positions, planting areas and crop years. On this basis, we [38] proposed a tobacco leaves substitute scheme in tobacco blend maintenance, and the results showed that this substitute scheme could achieve artificial substitute level. In this work, we further extended previous investigations and introduced the SVM algorithm to the thermogravimetric analysis for the first time. Using TGA data as the information source, we demonstrated that auto-classification of tobacco planting area and growing position could be achieved with high accuracy as well as high efficiency by applying the SVM algorithm. In view of the high similarity between tobacco and other biomasses in the compositions and pyrolysis behaviors, this new protocol, which couples the TGA data with the SVM algorithm, can potentially be extrapolated to the auto-classification of other biomass types.

## Results and discussion

### Classification of tobacco leaves

Eighty-eight tobacco leaves were collected from different growing positions in Fujian (FJ) and Yunnan (YN) provinces, which are shown in Table 1. Eighty-eight single-grade tobacco leaves were classified into eight categories according to their planting areas and growing positions. Three positions are identified, namely B, X, and C, corresponding to the upper, lower, and middle positions of tobacco stalk, respectively. The middle group is further divided into two subgrades, as shown in Table 1. The notation FJ-C1 implies that the sample is at the first grade of the middle group from the Fujian province.

**Table 1:** Categories of 88 single-grade tobacco leaves

Categories	Type^a,b^	Sample code
1	FJ-B	1–7
2	FJ-X	8–10
3	FJ-C1	11–20
4	FJ-C2	21–35
5	YN-B	36–44
6	YN-X	45–50
7	YN-C1	51–64
8	YN-C2	65–88

^a^FJ represents Fujian province and YN represents Yunnan province

^b^B, X, and C correspond to the upper, lower, and middle portions of tobacco stalk, respectively

Although all these samples, planted in either Fujian or Yunnan provinces, have similar tobacco style (all belonging to the same light-flavor style), they can still be distinguished in artificial sensory analysis. This leads to the most stringent test for the auto-classification of tobacco style to verify the effectiveness and practicability of the SVM model in the analysis of thermogravimetric analysis data.

### Analysis of thermogravimetric analysis data

**Fig. 1:**
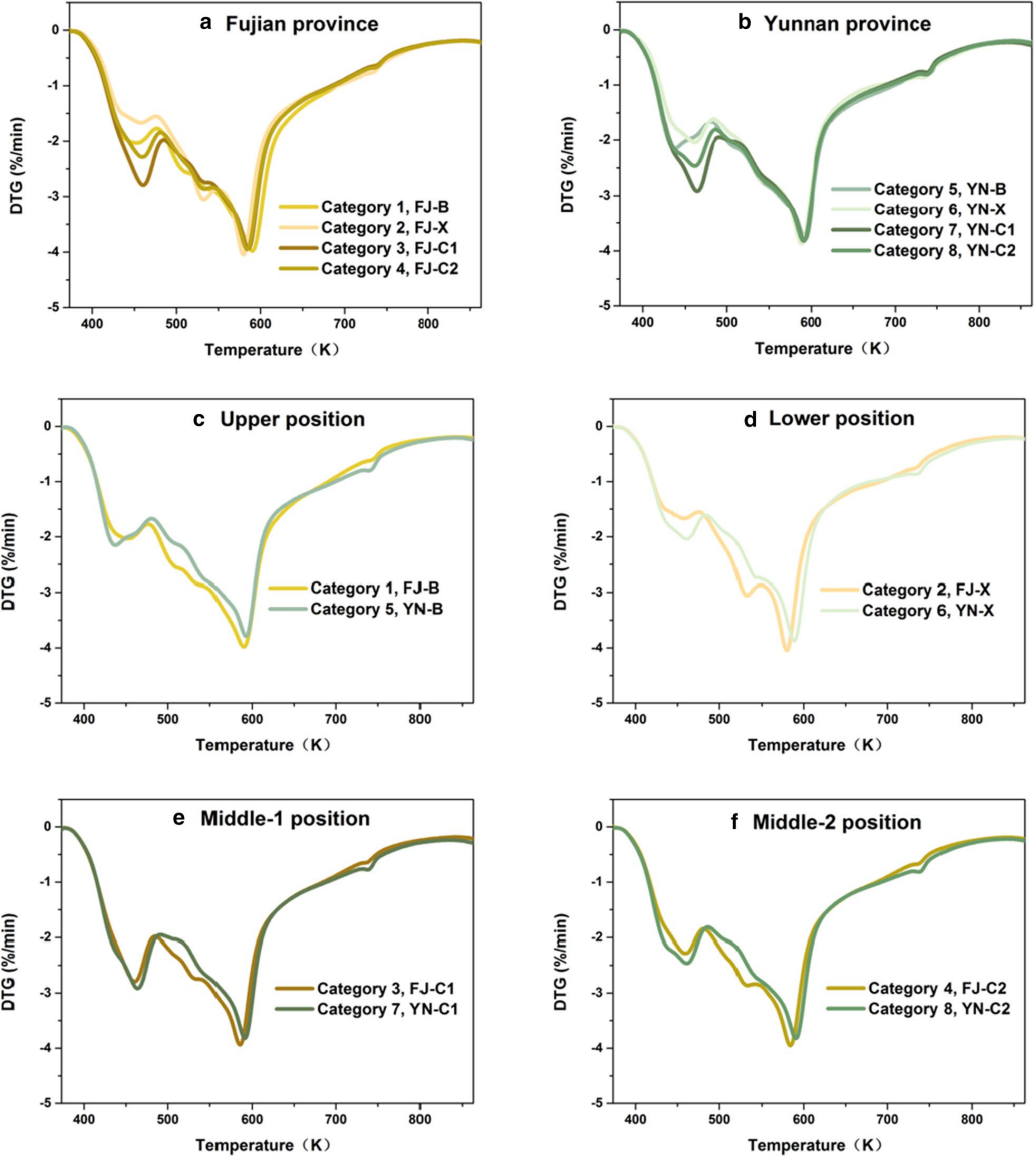
Comparison of the thermogravimetric analysis curves of tobacco leaves between eight categories. **a** The DTG curves of averaged four grades of Fujian province. **b** The DTG curves of averaged four grades of Yunnan province. **c** The DTG curves of averaged grade B from Fujian and Yunnan province. **d** The DTG curves of averaged grade X from Fujian and Yunnan province. **e** The DTG curves of averaged grade C1 from Fujian and Yunnan province. **f** The DTG curves of averaged grade C2 from Fujian and Yunnan province

For a better comparison, the thermogravimetric analysis data (DTG curves) of tobacco leaves belonging to the same category were averaged to obtain an averaged-DTG curve, which can represent the pyrolysis characteristics of the corresponding type of tobacco leaves, as shown in Fig. 1a, b.

Close analysis of Fig. 1a, b reveals that the main differences in the DTG curves of tobacco leaves from the same planting area lie in the temperature range of 373–473 K, which correspond to the thermal decompositions of sugar, nicotine, pectin, and some other volatile species. While in the temperature range of 473–873 K (corresponding to the pyrolysis of hemicellulose, cellulose and lignin), the DTG curves are basically coincident. Figure 1c–f presents the comparisons of DTG curves of tobacco leaves from the same growing position but from different planting areas. It is found that the main differences fall in the temperature range of 473–873 K. Hence, we may infer, from the thermogravimetric analysis spectra point of view, that the physical structure characteristics of tobacco leave (hemicellulose, cellulose, and lignin reflect the tobacco physical structure) is determined by the planting area. Namely, the tobacco leaves from the same planting area have similar physical structure characteristics, while the tobacco leaves from different planting areas have different physical structure characteristics. We may also draw the conclusion that the grade of tobacco leaves qualitatively depends on the proportion of sugar, nicotine, pectin, and some other volatile species, in which X < B < C2 < C1.

**Fig. 2:**
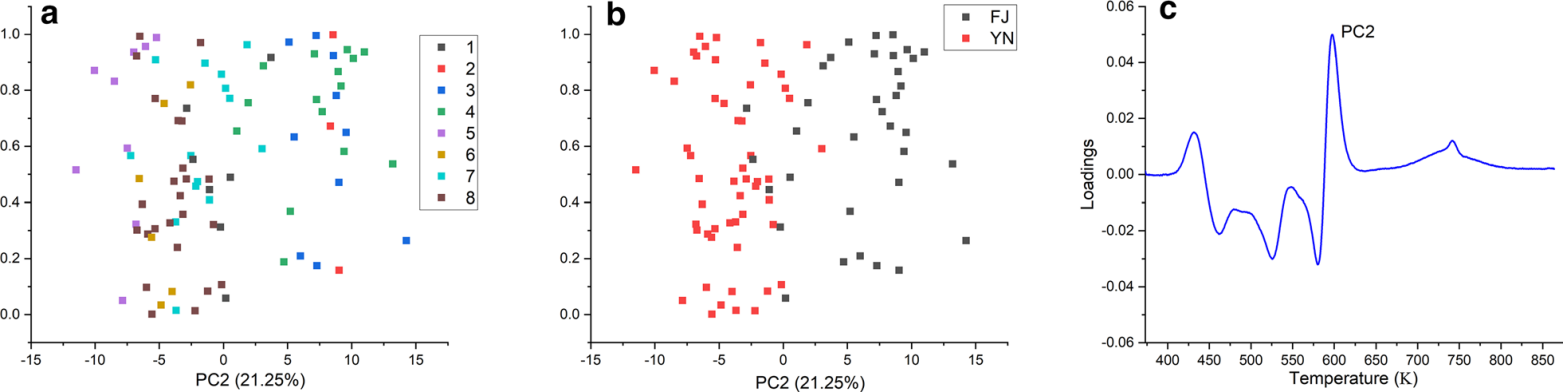
PCA analysis of the thermogravimetric analysis curves of 88 tobacco leaves. **a** Scores of 88 tobacco leaves in eight categories on the second principal component (PC2). **b** Scores of 88 tobacco leaves categorized by the planting areas on the second principal component (PC2). **c** Loadings of PC2 in the feature space

To further validate the above statement, we performed a principal component analysis (PCA) to study what spectral features characterize the different groups of samples (see Additional file 1 for other details). It is encouraging to see from Fig. 2c that the major contributions to the second principal component (PC2), which is mainly responsible for the classification of tobacco style (Fig. 2b), lie in the temperature range of 473–873 K. This result further strengthens the argument that the planting area characteristics of tobacco determined by the tobacco physical structure are mainly reflected in the temperature range of 473–873 K.

In summary, our preliminary analysis reveals that the growing position characteristics of tobacco, which is closely related to the content of sugar, nicotine, pectin, and some other volatile species, are mainly reflected in the temperature range of 373–473 K. The planting area characteristics of tobacco determined by the tobacco physical structure are mainly reflected in the temperature range of 473–873 K. These results are in line with how the traditional classification of tobacco leaves is performed in tobacco industry, namely the grade and style are discriminated separately.

### Algorithm

The above preliminary analysis has demonstrated that the thermogravimetric analysis data can reflect the planting area and growing position characteristics of tobacco leaves. To achieve auto-classification of tobacco leaves, machine learning is introduced to analyze the thermogravimetric analysis data.

Among numerical algorithms for machine learning, the traditional neural network algorithm requires a large amount of training data. However, due to sampling limitation, the number of samples (88) in this work cannot meet the requirements of neural networks for data training. Meanwhile, too many feature points (5890) in comparison to the number of samples (88) may also lead to dimensional disaster in neural network [39, 40]. For classification problems, the SVM algorithm [41] has been proven to be one of the best supervised learning algorithms, with faster speed and smaller sample size than other machine learning algorithms [42]. Therefore, we choose the SVM algorithm to perform auto-classification of tobacco quality and style. We would like to note that the traditional SVM algorithm only supports two categories, but our case involves eight different categories. Hence, the one-against-one method is adopted [43].

### Dataset sampling

Investigations on the generalization performance of SVM indicated that the sizes of the training set, validation set, and testing set are crucial for the estimated model performance [44]. Too many or too few samples in the training set may have a negative effect. Hence, it is necessary to have a good balance between the sizes of the training set and validation set to have a reliable estimation of model performance. Typically, one can take around 70–80% of the data to use as a training set and split the remaining data as the validation and testing set. In this work, 88 samples were split into three sets: training set, validation set, and testing set with ratio of 64/12/12, as shown in Table 2. Kennard-Stone-like algorithm [45] for data splitting was employed to maintain the generalization of the model. Namely, given n samples available in a category, the first m (with 0.6 < m/n and m <  = n) samples with largest Euclidian distance in this category are used as the training set and the unselected samples are randomly split into the validation and testing set with a ratio of 1/1.

**Table 2:** The sample codes for tobacco leaves of eight categories

Categories	Type^a,b^	Sample code		
		Training set	Validation set	Testing set
1	FJ-B	1–3, 6, 7	4	5
2	FJ-X	8–10		
3	FJ-C1	11, 13–18, 20	19	12
4	FJ-C2	21, 22, 24–27, 29, 30, 32, 33, 35	23, 28,	31, 34
5	YN-B	36–39, 42–44	41	40
6	YN-X	46, 48–50	47	45
7	YN-C1	53–56, 58–61, 63, 64	51, 52	57, 62
8	YN-C2	65–68, 70, 72, 74, 76, 77, 81–87	78, 79, 80, 88	69, 71, 73, 75

^a^FJ represents Fujian province and YN represents Yunnan province

^b^B, X, and C correspond to the upper, lower, and middle portions of tobacco stalk, respectively

### Model selection

**Fig. 3:**
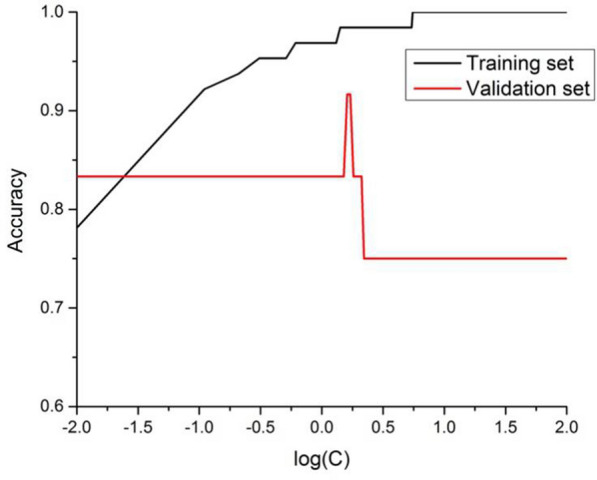
The influence of penalty parameter C on the accuracy of the training and validation set

Kernel function often plays an important role while classifying with SVM. Different kernel functions may have different application scopes. In the case where the number of feature points is much larger than the number of samples, the linear kernel has been proven to perform very well [46]. Hence, the linear kernel function was selected for training in this work. On the other hand, the penalty parameter C in the linear classification with SVM also plays a significant role in the training and prediction. Too small or too large C may have a negative effect on the prediction power of the model. To find an optimal C, the training set was used to build the model for each C and each trained model was tested with the validation set. As the samples in the validation set are not known to the model, therefore, the performance on the validation set can reflect the prediction power of the model. Based on the performance on the validation set, the optimal penalty parameter C was determined using the one with the highest accuracy. As shown in Fig. 3, the model has an excellent performance in both the training and the validation set when the penalty parameter C equals to 1.66 (Log(C) = 0.22), with accuracy being 98.44% and 91.67%, respectively. Therefore, the penalty parameter C was chosen to be 1.66 in this work.

### Classification accuracy

In the field of machine learning and the problem of classification with multiple categories, classification accuracy alone might be misleading. The confusion matrix can give a better idea of what the model is getting right and what types of errors it is making.

**Fig. 4:**
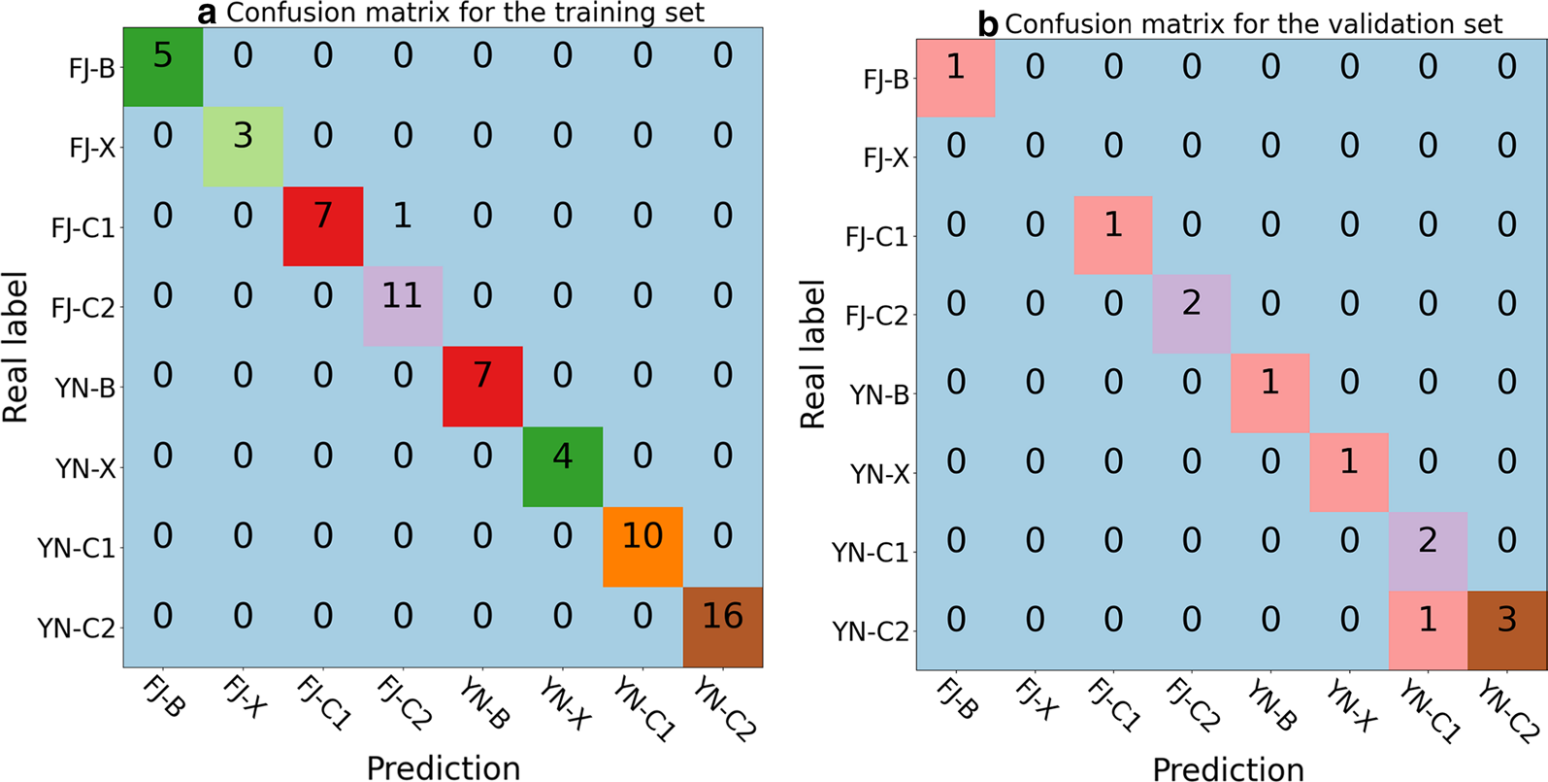
The confusion matrix for the training and validation set. The horizontal axis is the predicted label and the vertical axis is the real label

Detailed analysis of the performance of our optimal model on the training and validation set demonstrated that our optimal model performed remarkably well in the style classification, giving all correct results for the planting area, as shown in Fig. 4a, b. In the case of growing position classification, our model also correctly identified the upper, lower, and middle positions. Errors only occur in the classification of the subgrades of middle, namely C1 and C2. For the training set, only one sample (sample code: 18) belonging to FJ-C1 was mis-assigned to FJ-C2. A similar incorrect prediction was also found in the validation set, in which the sample (sample code: 79) belonging to YN-C2 was predicted to be YN-C1 instead. As elucidated in Sect. 3, both C1 and C2 grades correspond to the middle position of tobacco stalk and the grade difference is relatively small in comparison to the grade difference between X/B and C. This might be the reason for the mis-assignment of samples in C1 and C2. We would like to note that none of the previous investigations have ever tried to discriminate subgrades of the middle. Nonetheless, our optimal model showed excellent performance in both the training and validation set with overall accuracies being 98.44% and 91.67%, respectively.

**Fig. 5:**
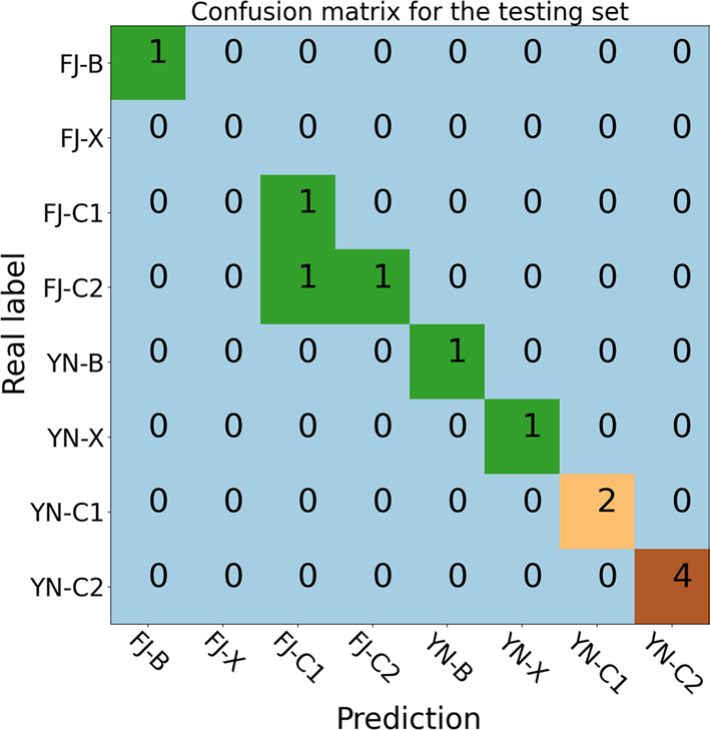
The confusion matrix for the testing set. The horizontal axis is the predicted label and the vertical axis is the real label

Westerhuis et al*.* [47] showed that the performance by cross-validation might be an over-optimistic one and it is of importance in having an additional blind test. To verify the practicality and generalization capability of our model, we further applied the optimal model to the testing set, which is not used during the model training and selection. It is found that this model works very well in the testing set, with an overall accuracy of 91.67%. One out of 12 samples was misclassified. Detailed analysis of the confusion matrix for the testing set, as shown in Fig. 5, indicated that our model performed extremely well in the prediction of planting areas as well as in the prediction of major grades of the upper, lower, and middle positions. None of the 12 samples was misclassified. Like in the training and validation set, the error only occurs in the classification of subgrades of the middle while applying our model to the testing set. The sample (sample code: 34) belonging to FJ-C2 was misclassified to FJ-C1). Such a high accurate blind test indicates that our model has an excellent generalization capability. We also applied the PLS-DA [48] to the same datasets. The optimal accuracy was found for the validation set with 25 latent variables (see Additional file 1 for details), and the corresponding accuracies for the training, validation, and testing set were 99.97%, 84.25%, and 82.78%, respectively. Compared to the PLS-DA algorithm, the SVM algorithm has a considerably higher classification accuracy.

It is worthwhile to note that both Fujian and Yunnan provinces locate in the south of China, and the tobacco styles of the two provinces are relatively close among the traditionally defined three major scent types, belonging to the light-flavor type. Previous investigations have demonstrated that the differences in the DTG curves between tobaccos planted in Fujian and Yunnan provinces are much smaller than those of the others [37]. It is encouraging to see that our SVM model, based on the thermogravimetric analysis spectra, still can achieve as high accuracy as 91.67% under such a stringent test, verifying the feasibility and practicability of the auto-classification of tobacco planting area and growing position. Unfortunately, we are unable to collect sufficient samples of other styles of tobacco at the current stage. We will leave them for the further investigation in future study.

It is well accepted that the biofuel compositions are near related to the biomass compositions and their pyrolysis behaviors [3–9]. Previous investigations on biomass have disclosed that the differences in pyrolytic characteristic are mainly caused by the difference in the constituent and physical structure [21–27]. Of particular note is that Enrico and Leonardo [23] have set up a simple and generalized procedure, which can be used for obtaining the chemical composition of lignocellulosic biomass based on their DTG curves. This procedure has been validated on 37 biomass types, such as woods, energy crops, and agricultural and food residues. Therefore, biomass types can be easily reflected on the DTG curves due to different chemical compositions and contents in it. Taking tobacco as a case study, we have shown in this work that a simple protocol, by coupling the TGA data with SVM algorithm, can be efficiently used for auto-classification of tobacco style and grades with high confidence. As a kind of lignocellulosic biomass, tobacco is different from other biomass types only in that its value is reflected in cigarettes via its pyrolysis reaction, and the content of each chemical composition is different. Hence, the protocol presented in this work can potentially be extrapolated to other biomass types.

## Conclusions

In this study, we conducted a thermogravimetric analysis over 88 single-grade tobacco leaves belonging to four grades and eight categories. Preliminary analysis of the thermogravimetric analysis spectra reveals that the tobacco leaves from the same planting area have similar physical structure characteristics, while the tobacco leaves from different planting areas have different physical structure characteristics, which are reflected in the DTG curves in temperature range of 473–873 K. Further analysis of the DTG curves also demonstrate that the growing position characteristic of tobacco leaves is mainly reflected in the temperature range of 373–473 K. On this basis, we introduced the SVM algorithm to automatically classify the planting area and growing position of tobacco leave using the thermogravimetric analysis spectra as the information source. This protocol, by coupling the DTG data with SVM algorithm, shows excellent performances in both the training and validation set as well as in the blind test, with overall accuracy over 91.67%. Throughout the whole dataset of 88 samples, our model not only provides precise results on the planting areas of tobacco leaves, but also accurately distinguishes major grades of the upper, middle, and lower parts of the tobacco stalk. The error only occurs in the classification of the subgrades of the middle. In the blind test, the sample (sample code: 34) belonging to FJ-C2 was misclassified to FJ-C1. Such a high accuracy in the blind test indicates that this protocol has an outstanding generalization capability. As a kind of lignocellulosic biomass, tobacco is different from other biomass types only in that its value is reflected in cigarette via its pyrolysis reaction, and the content of each chemical composition is different. Hence, the protocol presented in this work can potentially be extrapolated to other biomass types.

## Methods

### Materials

The tobacco samples were supplied by Fujian China Tobacco Industry Co., Ltd. For 48 h prior to analysis, all tobacco samples were conditioned in a chamber at 22 ± 1 °C and with a relative humidity of 60 ± 2%.

### Thermogravimetric analysis experiment

To guarantee the reproducibility, tobacco samples were pulverized into powder using a coffee mill and then sifted through a 100-mesh sieve to remove big tobacco particles before the TGA test.

Pyrolysis of tobacco powder was performed in a TGA (STA 449 F3 TG–DTA/DSC Instruments, NETZSCH, Germany). 10 mg of tobacco powder was loaded evenly in an open ceramic pan and warmed up to 873 K from room temperature at a heating rate of 10 K/min. Dry nitrogen at a flow rate of 100 mL/min was used as purge gas throughout the test. To reduce the influence of water, the thermogravimetric analysis data (DTG curve) of 373–873 K were selected for calculation and analysis. The number of feature points of each sample is 5890 which were obtained by recording 120 feature points per minute. The DTG curves of all 88 samples are given in Additional file 1.

### SVM

SVM algorithm is a linear classifier defined on the feature space to maximize the interval. It is essentially a convex optimization problem. Given the training data set in the feature space:

1 $$ \{ (x_{1} ,y_{1} ),(x_{2} ,y_{2} ),(x_{3} ,y_{3} ), \cdots ,(x_{n} ,y_{n} )\} , $$

where $${x}_{i}=({a}_{1}^{\left(i\right)},{a}_{2}^{\left(i\right)},{a}_{3}^{\left(i\right)},\cdots ,{a}_{m}^{\left(i\right)})$$ and $${y}_{i}\in \left\{-\mathrm{1,1}\right\}.$$

The training procedure in SVM is to find a hyper-plane, denoted as decision boundary, in the feature space, which can maximize the separation, namely margin, of samples in different classes. This hyper-plane is described as:

2 $$ w \cdot x + b = 0, $$

where *w* is the slope and *b* is the intercept.

Assuming that the training data set is linearly separable, there are infinitely separated hyper-planes. Linearly separable support vector machine (LS-SVM) solves the hyper-plane by maximizing the margin, and the solution is unique.

**Fig. 6:**
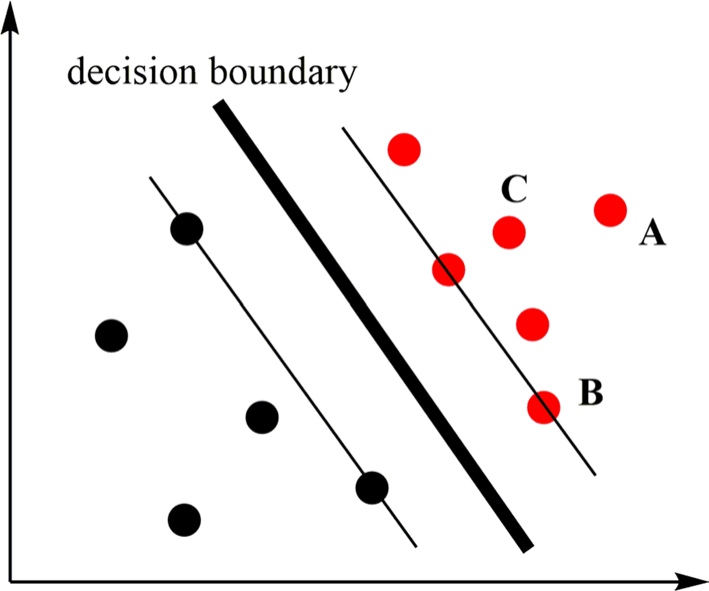
The points in the feature space, the supporting vectors, and the decision boundary (bold)

The distance between the points in the feature space and the decision boundary can represent the confidence level of the classification results, as shown in Fig. 6. Point A is far from the decision boundary, so the confidence of classification result is high. Point B is close to the decision boundary, so the confidence of classification result is low. Point C is between A and B, and the confidence of classification results is between that of two points.

The distance from the Point *x*_*i*_ to the decision boundary $w \cdot x + b = 0$ is the magnitude of $\hat{y}_{i} = w \cdot x_{i} + b$. Then, the function interval is described as follows:

3 $$ \hat{\gamma }_{i} = y_{i} \left( {w \cdot x_{i} + b} \right). $$

If *w* and *b* were changed in an equal proportion, though the position of the decision boundary will not change, the function interval would be changed correspondingly. Therefore, the geometric interval is introduced as follows:

4 $$ \gamma_{i} = y_{i} \left( {\frac{w}{\left\| w \right\|} \cdot x_{i} + \frac{b}{\left\| w \right\|}} \right). $$

To solve SVM is to find the decision boundary which maximize the geometric interval of points in feature space:

5 $$ \mathop {\max }\limits_{w,b} \mathop {\min }\limits_{i} \gamma_{i} \;\;\; $$

6 $$ {\text{s.t.}}\;\;y_{i} (w \cdot x_{i} + b) \ge 1,\;i = 1,2,...N. $$

This nonlinear optimization with inequality constraints can be further reduced to:

7 $$ \mathop {\max }\limits_{w,b} \;\;\;\frac{2}{{\left\| w \right\|^{2} }}\;\; \Leftrightarrow \;\mathop {\min }\limits_{w,b} \;\;\frac{1}{2}\left\| w \right\|^{2} $$

8 $$ s.t.\;\;\;\;\;y_{i} \left( {w \cdot x_{i} + b} \right) \ge 1,\;i = 1,2, \ldots ,N. $$

Then, the geometric interval of points in space from the decision boundary is used as the decision value, whose sign determines the result of classification.

However, in the case where the training data set is linearly non-separable, some sample points cannot satisfy the constraint condition (Eq. 8). A relaxation variable $\xi_{i}$ can be introduced to make the function interval with the relaxation variable greater than or equal to 1. Then, the previous optimization problem transforms into:

9 $$ \mathop {\min }\limits_{w,b,\xi } \;\;\frac{1}{2}\left\| w \right\|^{2} + C\sum\limits_{i = 1}^{N} {\xi_{i} } \;\; $$

10 $$ \begin{gathered} s.t.\;\;\;\;\;y_{i} \left( {w \cdot x_{i} + b} \right) \ge 1 - \xi_{i} ,\;\;i = 1,2, \cdots ,N \hfill \\ \;\;\;\;\;\;\;\;\xi_{i} \ge 0,\;\;i = 1,2, \cdots ,N, \hfill \\ \end{gathered} $$

where *C* (> 0) is the penalty parameter, which controls the trade-off between minimizing the training error $\sum\nolimits_{i = 1}^{N} {\xi_{i} }$ and maximizing the classification margin. Large values of C minimize the margin’s width of SVM and increase the weight of the non-separable samples. And, with a small value of C, the margin width was maximized, and the misclassified samples were increased. Optimal C can be obtained by applying grid search to find the value that achieves the maximum classification accuracy on the validation set.

The SVM model was trained with Scikit-learn (version 0.23) [49] in python 3.7 and original data from the thermogravimetric analysis experiment were adopted.

## Supplementary Information

**Additional file 1: Figure S1.** Thermal analysis curves of tobacco leaves of 8 categories. **Figure S2.** Scores of 88 tobacco leaves in eight categories on the first principal component (PC1). **Figure S3.** Scores of 88 tobacco leaves categorized by the planting area on the first principal component (PC1). **Figure S4.** Scores of 88 tobacco leaves in eight categories on PC1 and PC2. **Figure S5.** Loadings of PC1 and PC2 in the feature space. **Figure S6. **Explained variance of principal components in PCA analysis. **Figure S7.** Dependence of accuracies on the number of latent variables for the training and validation set in the PLS-DA analysis.

## Data Availability

All data generated and analyzed in this study are included in this published article.
