# Supplementary material for: Auto-classification of biomass through characterization of their pyrolysis behaviors using thermogravimetric analysis with support vector machine algorithm: case study for tobacco
Source: Biotechnol Biofuels. 2021 Apr 27;14:106. doi: 10.1186/s13068-021-01942-w (PMC8077845; doi:10.1186/s13068-021-01942-w)
Supplement: Supplementary file 1 — Additional file 1: Figure S1. Thermal analysis curves of tobacco leaves of 8 categories. Figure S2. Scores of 88 tobacco leaves in eight categories on the first principal component (PC1). Figure S3. Scores of 88 tobacco leaves categorized by the planting area on the first principal component (PC1). Figure S4. Scores of 88 tobacco leaves in eight categories on PC1 and PC2. Figure S5. Loadings of PC1 and PC2 in the feature space. Figure S6. Explained variance of principal components in PCA analysis. Figure S7. Dependence of accuracies on the number of latent variables for the training and validation set in the PLS-DA analysis. [file 13068_2021_1942_MOESM1_ESM.doc]

Auto classification of biomass through characterization of their pyrolysis behaviors by using thermogravimetric analysis with support vector machine algorithm: Case study for tobacco

Chao Yin^1^, Xiaohua Deng^2^, Zhiqiang Yu^2^,Zechun Liu^2^, Hongxiang Zhong^2^, Ruting Chen^1^, Guohua Cai^2^, Quanxing Zheng^2^, Xiucai Liu^2^, Jiawei Zhong^2^, Pengfei Ma^2^, Wei He^2^, Kai Lin^2^, Qiaoling Li^*2^, Anan Wu*[[1]](#footnote-2)


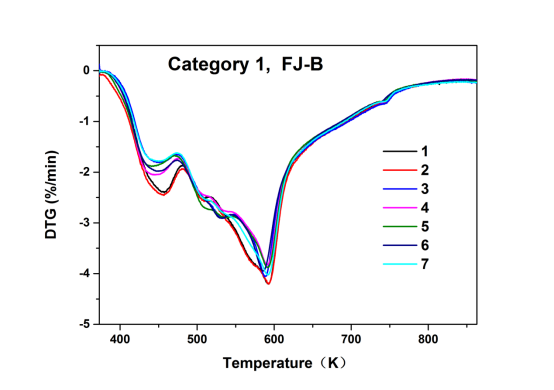

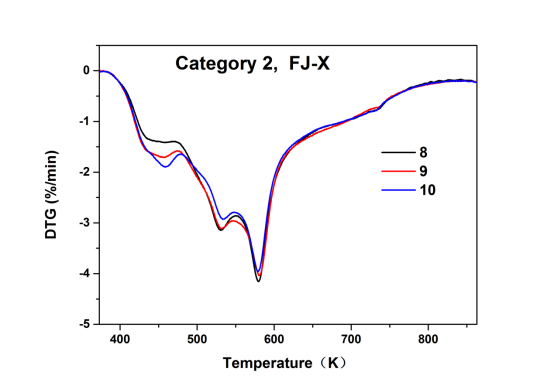

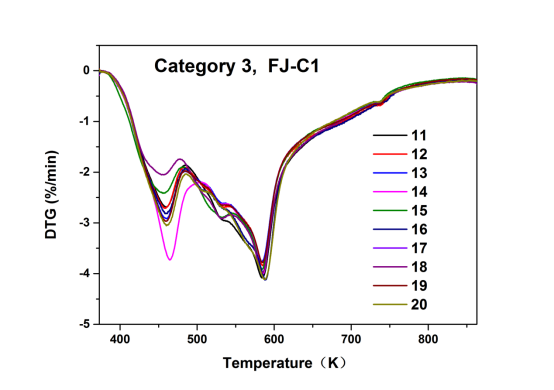

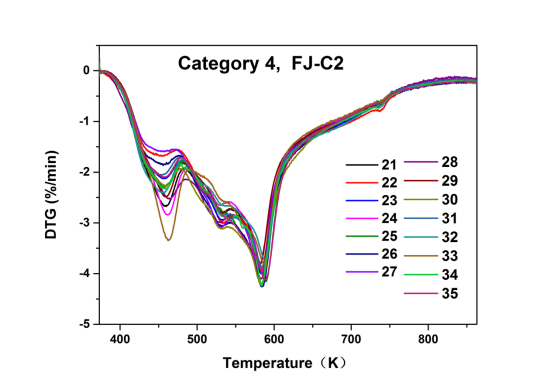

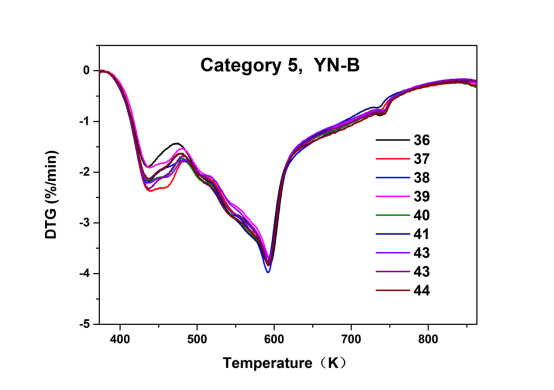

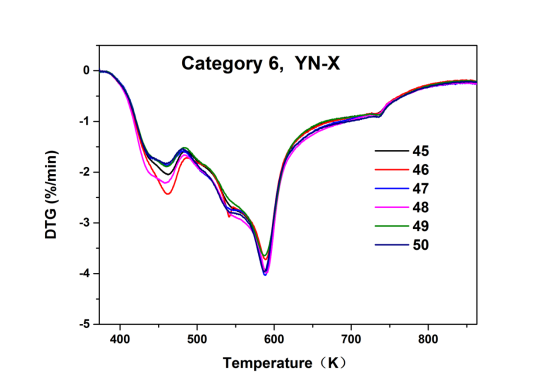

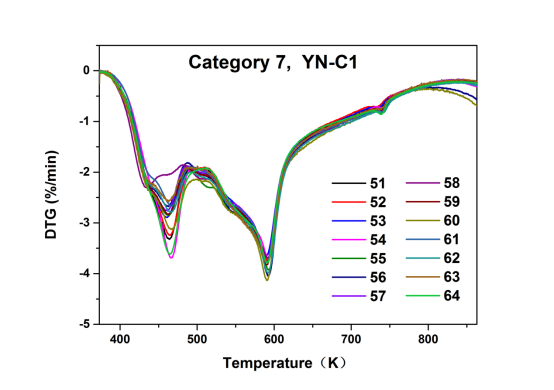

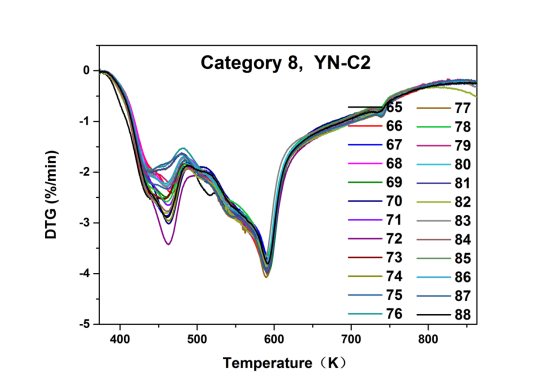


**Figure S1**. Thermal analysis curves of tobacco leaves of 8 categories.





**Figure S2**. Scores of 88 tobacco leaves in eight categories on the first principal component (PC1).


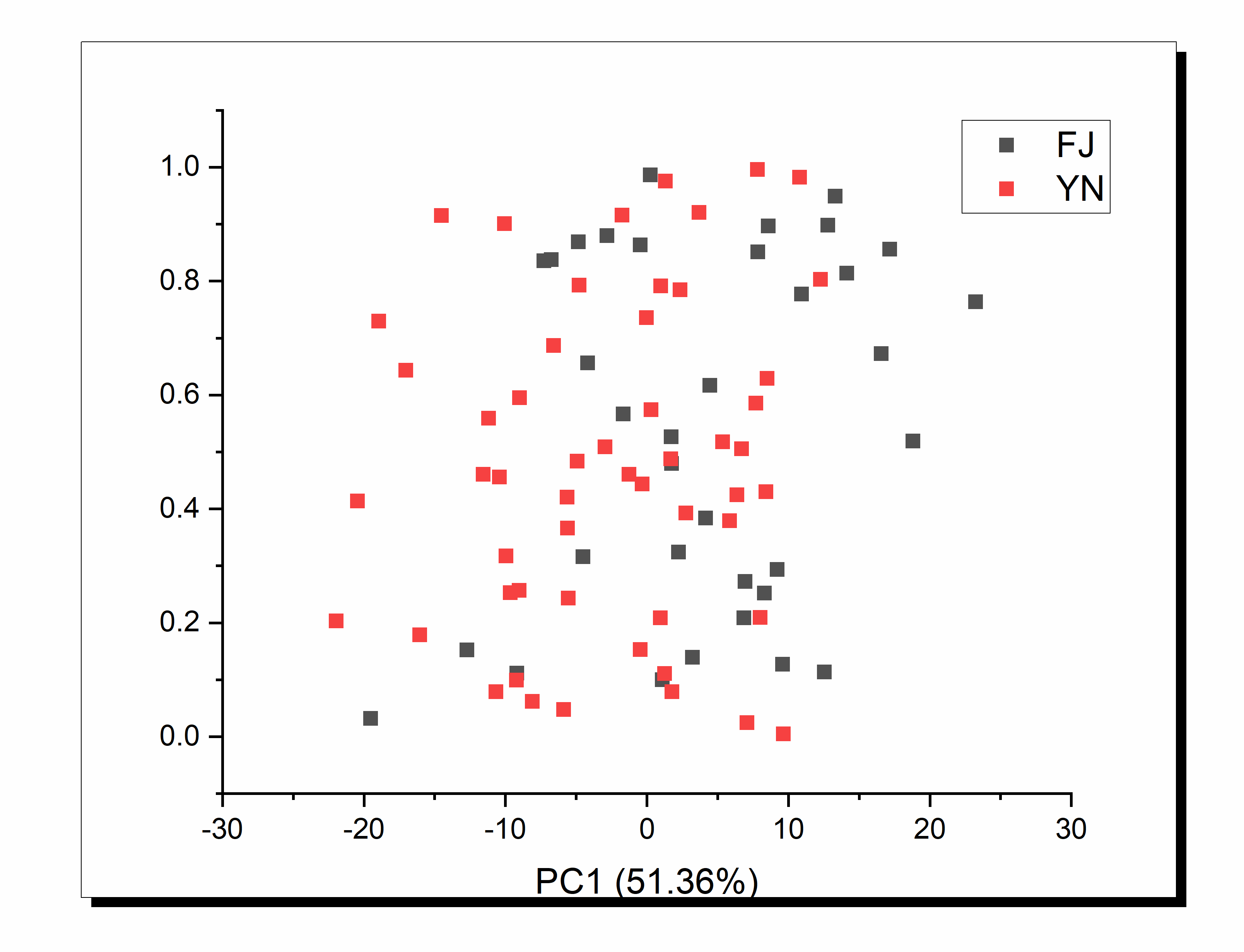


**Figure S3.** Scores of 88 tobacco leaves categorized by the planting area on the first principal component (PC1).





**Figure S4**. Scores of 88 tobacco leaves in eight categories on PC1 and PC2





**Figure S5**. Loadings of PC1 and PC2 in the feature space





**Figure S6**. Explained variance of principal components in PCA analysis.





**Figure S7**. Dependence of accuracies on the number of latent variables for the training and validation set in the PLS-DA analysis.

1. * Correspondence: [lql10684@fjtic.cn](mailto:zhx10411@fjtic.cn); ananwu@xmu.edu.cn

   1. Fujian Provincial Key Laboratory for Theoretical and Computational Chemistry, College of Chemistry and Chemical Engineering, Xiamen University, Xiamen 361005, Fujian, China

   2. Technology Center, China Tobacco Fujian Industrial Co., Ltd., Xiamen 361021, Fujian, China

   Full list of author information is available at the end of the article [↑](#footnote-ref-2)
